# Supplementary material for: Biglycan Alleviates Age-Related Muscle Atrophy and Hepatocellular Senescence
Source: Int J Mol Sci. 2025 Aug 26;26(17):8286. doi: 10.3390/ijms26178286 (PMC12428266; doi:10.3390/ijms26178286)
Supplement: Supplementary file 1 [file ijms-26-08286-s001.zip › ijms-3805164-supplementary.pdf]

Supplementary Materials

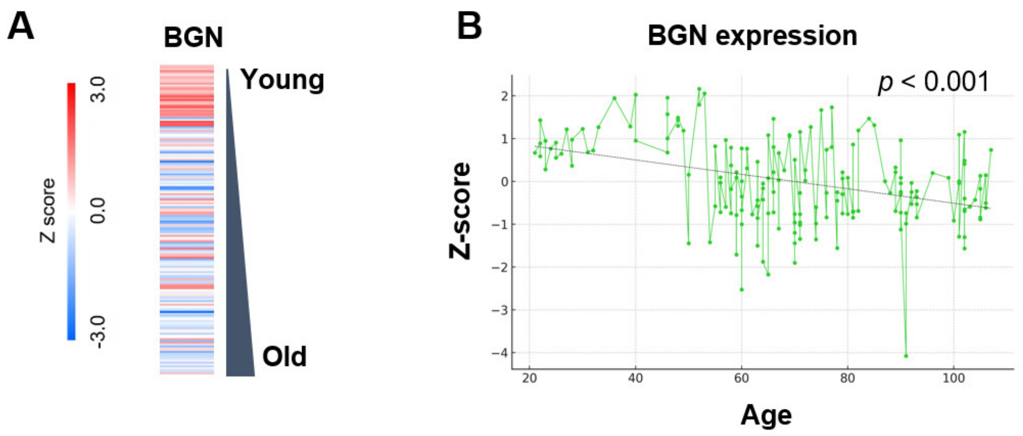

Supplementary Figure S1. The circulating level of biglycan declines with age. Plasma proteomic profiles from human aged 21 to 107 years were analyzed, and the change of biglycan in plasma with age was determined and z-scored for visualization as heatmap (A) and line plot (B).

Supplementary Table S1: List of primers for specific genes of mouse

Gene	Forward (5'- 3')	Reverse (5'- 3')
BGN	AGTGGCTTTGAACCAGGAGC	AGTAGGTCCTCCAACTCAATAG
p16	CCCAACGCCCCGAACT	GCAGAAGAGCTGCTACGTGAA
p21	AACATCTCAGGGCCGAAA	TGCGCTTGGAGTGATAGAAA
IL6	TAGTCCTTCCTACCCCAATTTCC	TTGGTCCTTAGCCACTCCTTC
TGFβ1	CTCCCGTGGCTTCTAGTGC	GCCTTAGTTTGGACAGGATCTG
Foxo3	AAACGGCTCACTTTGTCCCA	TTGTGCCGGATGGAGTTCTTC
4EBP1	CCTCCTTGTGCCTCTGTCTA	GCCTAAGGAAAGATGGGTGT
Atrogin	ATGCACACTGGTGCAGAGAG	TGTAAGCACACAGGCAGGTC
MuRF1	CTGGAGGTCGTTTCCGTTGC	TCGGGTGGCTGCCTTTCTGC
Myogenin	CTACAGGCCTTGCTCAGCTC	TGTGGGAGTTGCATTCACTG
MyHC	TCCAAACCGTCTCTGCACTGT	AGCGTACAAAGTGTGGGTGTG
Mtsn	TAACCTTCCCAGGACCAGGA	CACTCTCCTGAGCAGTAATT
KLF15	GGCAGTGGAGGTATTGGAGAT	GGTCCCTGCTACCGTTCTCT
Hspb7	TGTCACCACCTTCAACAACCAC	TCATGACTGTGCCATCAGCTG
TNFα	AAGCCTGTAGCCCACGTCGTA	GGCACCACTAGTTGGTTGTCTTTG
CD36	TGCACCACATATCTACCAAA	TTGTAACCCCACAAGAGTTC
GPAT	GTAGTTGAACTCCTCCGACA	ATCCACTACCACTGAGAGGA
Lipin1	TGCAGTTTGTGAACGAGGAG	TGGAAGGGGAATCTGTCTTG
Nqo1	AGAGAGTGCTCGTAGCAGGAT	GTGGTGATAGAAAGCAAGGTCTT
HO-1	GATAGAGCGCAACAAGCAGAA	CAGTGAGGCCCATACCAGAAG
CAT	TGGCACACTTTGACAGAGAGC	CCTTTGCCTTGGAGTATCTGG
L32	GGCCTCTGGTGAAGCCCAAGATCG	CCTCTGGGTTTCCGCCAGTTTCGC
Hprt	AAGCTTGCTGGTGAAAAGGA	TTGCGCTCATCTTAGGCTTT
Gapdh	CACCATCTTCCAGGAGCGAG	CCTTCTCCATGGTGGTGAAGAC
